# Surgery to chemoradiotherapy time may not impact outcomes in glioblastoma patients treated with modern techniques: a single-institution study

**DOI:** 10.2478/raon-2025-0031

**Published:** 2025-05-14

**Authors:** Volkan Semiz, Hasan Oguz Cetinayak, Barbaros Aydin, Cenk Umay, Fadime Can

**Affiliations:** 1 Department of Radiation Oncology, Faculty of Medicine, Dokuz Eylul University, Izmir, Turkey; 2 Department of Radiation Oncology, Izmir City Hospital, Izmir, Turkey

**Keywords:** glioblastoma, radiotherapy, treatment interval

## Abstract

**Background:**

Surgery followed by chemoradiotherapy (CRT) with temozolomide is the standard treatment for glioblastoma patients. But, the time between surgery and CRT is still a controversial issue. This study investigated the impact of delay in CRT after surgery on overall (OS) and progression-free survival (PFS).

**Patients and methods:**

Patients aged ≥ 18 years with IDH-wild type glioblastoma, who received 60 Gy concomitant CRT with temozolomide were included in the study. Exclusion criteria include patients who underwent biopsy only, had an Eastern Cooperative Oncology Group (ECOG) performance status > 1, or presented with multicentric tumors. The interval between surgery and CRT was categorized according to 42 days, and delays after this point were defined as delayed treatment initiation. Statistical analyses included Kaplan-Meier survival analysis and Cox regression models.

**Results:**

The median OS for the regular and delayed groups was 18 and 19 months, and the PFS was 11.8 and 14.6 months, respectively. Delayed patients showed better PFS, but no statistically significant difference was found between the groups in terms of OS and PFS (p = 0.149, p = 0.076). In multivariate analysis, ECOG performance score 1 and subtotal resection were associated with poor prognosis for both OS and PFS (for OS p = 0.031, p < 0.001; for PFS p = 0.038, p = 0.029). When the time from surgery to CRT was analyzed according to the extent of surgery, no significant difference was observed in OS and PFS (p = 0.068, P = 0.057).

**Conclusions:**

Our findings showed that delays of more than 42 days in adjuvant CRT did not affect OS or PFS. However, further studies are needed to evaluate the effects of delayed adjuvant therapy in patients with subtotal resection.

## Introduction

Glioblastoma (GB) poses a formidable challenge in neuro-oncology, characterized by a dismal prognosis despite multimodality treatment.^1^ The standard of care encompasses surgical resection followed by concurrent chemoradiotherapy (CRT) with temozolomide (TMZ) and subsequent TMZ monotherapy. While this regimen has improved outcomes, median survival remains limited, typically ranging from 12 to 18 months.

The interval between surgical resection and the adjuvant therapy emerges as a critical prognostic factor in various malignancies. Delays in starting CRT following surgery can influence treatment response rates and disease progression due to the proliferation of biologically active residual tumor cells. Therefore, as the interval between surgery and adjuvant treatment lengthens, local control and survival rates tend to decrease. However, studies aiming to determine the optimal timing for adjuvant therapy initiation in glioblastoma are limited and present heterogeneous results.^2,3^ Many studies report the optimal time between surgery and radiotherapy (RT) as 4–6 weeks.^4,5^

The extent of surgical resection stands out as a critical factor in GB treatment. Studies have shown that achieving the widest possible surgical resection can significantly increase survival rates.^6^ Especially in patients who undergo subtotal resection, adjuvant CRT is desired to be started as soon as possible due to the risk of rapid proliferation of residual tumor cells. However, various factors, including postoperative complications, delayed wound healing, logistical challenges in accessing radiotherapy facilities, and the evolving diagnostic landscape with the incorporation of more extensive immunohistochemical analyses for the 2021 World Health Organization (WHO) classification, can contribute to delays in initiating adjuvant therapy. The potential impact of these delays on disease progression warrants further investigation.

In this study, the effect of prolonged intervals between surgery and CRT on overall survival (OS) and progression-free survival (PFS) will be investigated in GB patients treated with modern radiotherapy techniques and concurrent TMZ. The findings obtained can contribute to determining optimal strategies in the planning of surgery and adjuvant treatment, potentially improving clinical outcomes in the management of GB patients.

## Patients and methods

This retrospective study included patients diagnosed with GB who underwent treatment at the radiation oncology department of our institution between 2015 and 2022. Inclusion criteria encompassed patients aged ≥ 18 years who underwent surgical resection, received radiotherapy with a total dose of 60 Gy delivered using intensity-modulated radiotherapy (IMRT) with volumetric modulated arc therapy (VMAT), and were administered concurrent TMZ. Patients were re-classified according to the 2021 WHO classification, and those previously diagnosed with IDH mutant GB were excluded from the study. Exclusion criteria included patients who underwent biopsy only, had an Eastern Cooperative Oncology Group (ECOG) performance status > 1, or presented with multicentric tumors. Patients with an ECOG performance status > 1 were excluded due to their tendency to start treatment earlier, which could bias the survival analysis. This study was approved by the Institutional Review Board (no: 2022/12-18).

All patients underwent surgical resection. The extent of resection was categorized as gross-total resection (GTR) or subtotal resection (STR) based on the neurosurgeon’s assessment and, when available, brain magnetic resonance imaging (MRI) performed within 72 hours postoperatively. In the pathological examination of the cases, the diagnosis was generally made with morphological and immunohistochemical findings. Molecular examinations were performed in the necessary cases. Histopathological findings such as hypercellularity, microvascular proliferation, increased mitosis and palisaded necrosis were observed in these glial tumors. No IDH-1 (R132) mutation was observed in the tumors immunohistochemically. All patients were evaluated with multiparametric MRI (contrast-enhanced brain MRI, diffusion MRI, perfusion MRI, and MR spectroscopy) in the 3^rd^–4^th^ weeks post-surgery, and the RT plan was made. All patients received concurrent TMZ according to the Stupp protocol.^7^ Following the completion of CRT, suitable patients received adjuvant TMZ monotherapy.

In the postoperative multiparametric MRI, the contrast-enhanced area, operation cavity, and areas suspected of residual tumor were defined as the gross tumor volume (GTV). According to our clinic’s protocol, clinical target volumes (CTV) were created with a 1 and 2 cm margin around the GTV, named CTV1 and CTV2, respectively. Planned target volumes (PTV) were then created with a 2 mm margin, with PTV1 receiving 60 Gy and PTV2 receiving 50 Gy RT in 30 fractions using simultaneous boost with IMRT-VMAT.

Patients were followed up with regular multiparametric MRI scans for disease progression. Imaging was performed every 3 months for the first 2 years and every 6 months thereafter. Progression was assessed according to the Response Assessment in Neuro-Oncology (RANO) criteria.^8^ Patients who died without progression at their last imaging were considered progression-free.

The time interval between surgery and the radiotherapy was defined as the duration from the date of surgery to the first day of radiotherapy. This time was evaluated by separating it according to the 42^nd^ day based on data from other studies and patients starting CRT after 42 days classified as delayed. Since the number of patients starting treatment before 28 days was too low, they were not analyzed as a separate group.

### Statistical analysis

OS was defined as the time from surgery to death, while PFS was defined as the time from surgery to progression. Statistical analyses were conducted using IBM SPSS v.29. Continuous variables were analyzed using the t-test, and categorical variables were analyzed using Pearson’s chi-square test or Fisher’s exact test. Survival analysis was performed using the Kaplan-Meier method and log-rank test, while univariate and multivariate analyses were conducted using Cox regression analysis. A p-value < 0.05 was considered statistically significant.

## Results

### Patient characteristics

A total of 91 patients who met the inclusion criteria were included in the study. The median age was 58 years (22-79). Postoperative CRT started at a median of 39 days (18-98). All patients received concurrent TMZ; however, 10 patients could not complete concurrent TMZ due to side effects. After CRT, patients received a median of 7 cycles (2–18) of adjuvant TMZ. Four patients could not receive adjuvant TMZ due to toxicity. The demographic and treatment characteristics of the patients are presented in Table 1.

**TABLE 1.** Patient characteristics

	All patients (n = 91)	< 42 days (n = 56)	≥ 42 days (n = 35)
Surgery to CRT, median (range), days	39 (18–98) days	34 (18–41) days	48 (42–98) days	
Age, median (range), years	58 (22–79)	59 (22–77)	58 (27–79)	p = 0.798
Gender				
Male	54 (59.3%)	31 (55.4%)	23 (65.7%)	p = 0.328
Female	37 (40.7%)	25 (44.6%)	12 (34.3%)	
ECOG Score				
0	63 (69.2%)	36 (64.3%)	27 (77.1%)	p = 0.246
1	28 (31.8%)	20 (35.7%)	8 (12.9%)	
Extent of Surgery				
GTR	64 (70.3%)	40 (71.4%)	24 (68.5%)	p = 0.816
STR	27 (29.7%)	16 (28.6%)	11 (31.5%)	
Adjuvant temozolomide cycles, median	7 (2–18) cycles	7 (2–18) cycles	7 (3–15) cycles	p = 0.385
PTV volumes, mean	186.7 cm^3^	187.8 cm^3^	185.2 cm^3^	p = 0.888

1 CRT = chemoradiotherapy; ECOG = Eastern Cooperative Oncology Group; GTR = gross-total resection; PTV = planned target volume; STR = subtotal resection

In the group starting treatment on time, the median interval between surgery and RT was 34 days, whereas it was 48 days in the delayed group. No differences were observed between the patient groups in terms of age, extent of surgery, performance score, number of adjuvant TMZ cycles, or PTV volume (Table 1).

### Survival analysis

**FIGURE 1.**
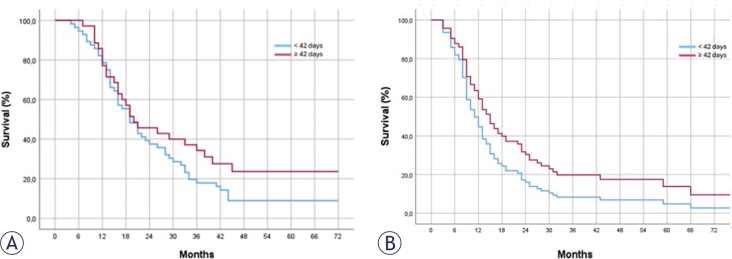
Overall **(A)** and Progression Free Survival **(B)**

The median OS and PFS of the entire group were 18.5 (95% CI: 15.4–20.5) and 13 months, respectively. According to the surgery-RT interval, the median OS for the regular and delayed groups was 18 (95% CI: 13.8–22.2) and 19 (95% CI: 9.7–28.3) months, PFS was 11.8 (95% CI: 8.4–13.6) and 14.6 (95% CI: 8.6–19.4) months, respectively. One-year OS rates were 75% (95% CI: 66.1–83.9) and 71% (95% CI: 61.7–80.3) for the regular and delayed groups, while PFS rates were 45% (95% CI: 34.8–55.2) and 58% (95% CI: 47.9–68.1), respectively (Figure 1). Although delayed patients showed better PFS, no statistically significant difference was found between the groups in terms of OS and PFS in both univariate (UVA) and multivariate analyses (MVA) [(in UVA: p = 0.161 HR:0.714 (95% CI: 0.446–1.143), p = 0.076 HR: 0.652 (95% CI: 0.406–1.046); in MVA: p = 0.060 HR:0.610 (95% CI:0.368–1.013), p = 0.071 HR:0.643 (95% CI:0.398–1.039)].

Other factors affecting OS in univariate analysis included an ECOG performance score of 1 (p = 0.018, HR 1.783 [95% CI: 1.103–2.881]) and subtotal surgical resection (p < 0.001, HR 2.304 [95% CI: 1.422–3.733]), both associated with poor prognosis. Multivariate analysis confirmed performance score (p = 0.031, HR 1.791 [95% CI: 1.056–3.037]) and surgical resection type (p < 0.001, HR 2.921 [95% CI: 1.702–5.014]) as predictors of poor prognosis.

For PFS, univariate analysis identified an ECOG performance score of 1 (p = 0.023, HR 1.805 [95% CI: 1.026-2.846]) and subtotal surgical resection (p = 0.007, HR 2.017 [95% CI: 1.219–3.337]) as predictors of poor prognosis. Multivariate analysis confirmed performance score (p = 0.038, HR 1.765 [95% CI: 1.032–3.019]) and surgical resection type (p = 0.029, HR 1.793 [95% CI: 1.063–3.025]) as significant factors. Prognostic factors affecting survival are shown in Tables 2 and 3.

**TABLE 2.** Univariate and multivariate Cox regression analysis for overall survival

	Univariate analyse		Multivariate analyse	
	HR (95% CI)	p value	HR (95% CI)	p value
**Age**				
**≤ 55**	REF 1.284 (0.812–2.031)	0.284	REF 1.535 (0.957–2.462)	0.076
**> 55**				
**Gender**				
**Male**	REF 0.874 (0.555–1.375	0.560	REF 0.819 (0.498–1.345	0.430
**Female**				
**ECOG score**				
**0**	REF 1.783 (1.103–2.881	**0.018**	REF 1.791 (1.056–3.037)	**0.031**
**1**				
**Extent of Surgery**				
**GTR**	REF 2.304 (1.422–3.733)	**< 0.001**	REF 2.921 (1.702–5.014)	**< 0.001**
**STR**				
**Surgery to CRT**				
**< 42 days**	REF 0.714 (0.446–1.143)	0.161	REF 0.610 (0.368–1.013)	0.060
**≥ 42 days**				

1 CRT = chemoradiotherapy; ECOG = Eastern Cooperative Oncology Group; GTR = gross-total resection; HR = hazard ratio; STR = subtotal resection

**TABLE 3.** Univariate and multivariate Cox regression analysis for progression free survival

	Univariate Analyse		Multivariate Analyse	
	HR (95% CI)	p value	HR (95% CI)	p value
**Age**				
**≤ 55**	REF 0.700 (0.445–1.101)	0.122	REF 0.701 (0.437–1.125)	0.141
**> 55**				
**Gender**				
**Male**	REF 0.915 (0.638–1.611)	0.915	REF 0.952 (0.591–1.534)	0.839
**Female**				
**ECOG Score**				
**0**	REF 1. 805 (1.026–2.846)	**0.023**	REF 1.765 (1.032–3.019)	**0.038**
**1**				
**Extent of Surgery**				
**GTR**	REF 2.017 (1.219–3.337)	**0.007**	REF 1.793 (1.063–3.025)	**0.029**
**STR**				
**Surgery to CRT**				
< 42 days	REF 0.652 (0.406–1.046)	0.076	REF 0.643 (0.398–1.039)	0.071
**≥ 42 days**				

1 CRT = chemoradiotherapy; ECOG = Eastern Cooperative Oncology Group; GTR = gross-total resection; HR = hazard ratio; STR = subtotal resection

After progression, 50 patients (54.9%) received second-line chemotherapy with Bevacizumab and Irinotecan, 15 patients (16.5%) underwent re-surgery, and 15 patients (16.5%) underwent re-irradiation.

Subgroup analysis was also conducted based on the extent of surgery. When patients were evaluated according to the performance score, which was identified as a factor influencing survival, a performance score of zero was observed in 16 patients (59%) who underwent subtotal resection (STR), whereas it was observed in 47 patients (73%) who underwent gross total resection (GTR) (p = 0.181). Patients undergoing STR had a median OS and PFS of 12 (95% CI: 10–13.9) and 10.8 (95% CI: 7.2–12.8) months for the regular group and 15 and 10.5 (95% CI: 5.1–14.9) months for the delayed group, respectively. In patients undergoing GTR, these durations were 23 (95% CI: 13.7–32.3) and 12 (95% CI: 7.5–14.6) months for the regular group and 20 (95% CI: 5.6–34.4) and 19 (95% CI: 8–27.9) months for the delayed group. When the surgery to CRT times were analyzed by surgical type, no significant differences in OS and PFS were detected (OS: p = 0.068, HR 0.633 [9%5 CI: 0.387–1.034]; PFS: p = 0.057, HR 0.625 [95% CI: 0.385–1.015]).

## Discussion

Glioblastoma is the most common primary brain tumor in adults, with low survival rates due to its aggressive nature. Numerous factors affect the survival in patients diagnosed with GB. Therefore, in our study, only patients with good performance were included, and patients diagnosed with IDH mutant GB according to former WHO classification but known to have a better prognosis were excluded. Additionally, there was no significant difference between the groups in terms of age and extent of surgical resection.

Due to the aggressive nature of glioblastomas, many centers aim to start adjuvant therapy soon after surgery. Several studies support that delayed adjuvant therapy reduces survival. Early studies, such as that by Burnet *et al*., reported a significant decrease in median survival with delayed RT.^9^ Similarly, Irwin *et al*. suggested that each week of delay in RT could reduce survival by 8.9%.^10^ However, these studies, which were performed before the TMZ era, have limitations, including limited use of concurrent chemotherapy, presence of grade 3 astrocytomas among patients and the delivery of RT doses below 60 Gy, which may not reflect current treatment paradigms.

Conversely, some studies have argued that shorter surgery-to-RT intervals reduce survival. An analysis of 16 RTOG trials involving 2855 patients in 2009 found that starting RT after four weeks significantly improved survival, while starting RT within two weeks reduced survival.^11^ However, this study did not use standard concurrent chemotherapy, and the patients were treated over a long period of time from 1974 to 2003. Therefore, the same group conducted a similar study in 2016 with 1385 patients, including concurrent TMZ. In this study, patients were categorized based on a four-week threshold, concluding that the surgery-to-RT interval did not affect survival. They attributed this to the use of concurrent TMZ, which they suggested played a more critical role than the surgery-to-RT interval and improved survival across all patient groups, making timing of RT less significant.^2^

Contrary to this, Nathan *et al*., in a study involving 2535 patients treated with the Stupp protocol, found that starting RT earlier than four weeks reduced survival, while initiating RT between six to thirteen weeks did not affect survival.^12^ However, because this study was planned as a database analysis, characteristics such as performance status of patients, extent of resection, tumor grade, and IDH mutation were unclear.

Although many studies recommend starting RT within 4–6 weeks after surgery, complications following surgery and the need for increasing immunohistochemical and molecular tests with the new WHO classification system for definitive diagnosis can prolong the surgery-to-RT interval.^13^ Additionally, in middle- and low-income countries, challenges in accessing radiotherapy centers or delays in imaging tests can extend this interval. In developed countries like the USA, nearly half of the patients start treatment within 4–8 weeks, while very few start after eight weeks.^14^ Consequently, some patients begin treatment after six weeks, raising concerns about their survival outcomes.

Zhang *et al*. reported decreased overall and progression-free survival in patients starting CRT after six weeks, with median survival decreasing from 26 months to 15 months.^15^ Yet, this study included a limited number of late-starting patients, most of whom were elderly, while the early-starting group included IDH mutant patients, who have better survival outcomes. Sun *et al*., using The Cancer Genome Atlas (TCGA) data, found that early commencement of RT did not affect survival, but starting RT after six weeks significantly reduced survival.^16^ In contrast, Press *et al*., using the National Cancer Database (NCDB) with 30 414 GB patients, reported that starting treatment after five weeks did not alter overall survival.^17^ However, because both trials relied on databases, there is a possibility of bias in patient selection. The RPA classification was employed in the Press *et al*. investigation, but additional variables that influence survival such as IDH mutation or extent of surgery, could not be assessed.

Several hypotheses have been proposed to explain the reduced survival with early postoperative RT. The most plausible is postoperative hypoxia. Hypoxia leads to increased expression of HIF-1α, which upregulates genes involved in tumor progression.^23^ Reduced blood flow to the residual tumor and surgical cavity postoperatively creates a hypoxic environment, which increases radioresistance.^24^ Initiating RT before blood flow improves may reduce treatment efficacy. Additionally, the surgical cavity shrinks significantly within the first four weeks post-surgery.^25,26^ A larger cavity in the early postoperative period can increase RT volumes and the volume of brain tissue receiving high-dose radiation. Animal models have shown that irradiation in the second postoperative week causes greater brain damage.^27^ This brain damage may delay patient recovery and reduce survival. Furthermore, clinicians may be inclined to expedite treatment in STR/biopsy cases or patients with poor performance scores, which could contribute to poorer survival outcomes due to the inclusion of worse-prognosis patients in the early-treatment group.^28^

The prolongation of the interval between surgery and RT in patients with STR remains another controversial issue. It is not known at what stage the prolongation of treatment will cause problems in patients who underwent STR. There are very few studies have addressed this matter. Ahn *et al*., in their study evaluating the impact of the surgery-to-RT interval on survival, reported that patients with partial resection who initiated treatment within four weeks had better survival, whereas no significant difference was observed in those who underwent gross total resection.^22^ In our study, the extension of the surgery to CRT period beyond six weeks based on the extent of surgery was evaluated, and no difference in survival was observed. However, it should be noted that this result may be misleading due to the small number of patients who underwent STR.

This study has some limitations. The retrospective nature of the study might have affected the results. The major limitation of study is the absence of MGMT status of patients. Apart from this, since the aim of our clinic is to start the treatment within 4-6 weeks, the number of patients with delayed CRT is low and this may underpower our analysis. In addition, the study evaluated only patients with good performance, which may not reflect the entire patient population well. But the literature reports that the percentage of patients with an ECOG score of 2 or higher ranges between 20–35%, so, we consider this a minor limitation in generalizing our findings to the entire population.^29^

As seen, the impact of the surgery-to-RT interval on survival has been debated for years with conflicting results (Table 4). Several large patient studies have evaluated this interval, but some relied on national databases where patient and treatment characteristics were not homogeneous or did not account for molecular features. While some of the studies included grade 3 astrocytoma, the majority of them did not take into account IDH status and poor performance score. Our study differs from others in that it excluded individuals with IDH mutations or low performance scores.

**TABLE 4.** Literature review of clinical impact of radiation delay following surgical resection

Study	Year	Patients	Cutoff points	TMZ	Median survival	Results
Irwin *et al*.^10^	2007	172	2 wks	-	7.8 mos in GB 14.9 mos for astrocytoma	Increased risk of death by 8.9% for each additional week
Noel *et al*.^18^	2012	400	6 wks	67%	13.4 mos	No differences in OS
Loureiro *et al*.^19^	2015	115	6 wks	60%	13.5 *vs*. 14.2 mos	No differences in OS
Sun *et al*.^16^	2015	218	6 wks	+	15.2 *vs*. 12.9 mos	Worse OS in > 6 wks
Wang *et al*.^20^	2016	447	3 wks	92%	12.3 *vs*. 15.3 mos	Worse OS in < 3 wks
Nathan *et al*.^12^	2017	2535	4–6 wks	+	21.3 *vs*. 26.6 *vs*. 27.6 mos	< 4 is associated with a 31% increased risk of death, no difference between > 4 *vs*. > 6
Blumenthal *et al*.^2^	2018	1395	4 wks	+	16 *vs*. 15.9 mos	No differences in OS and PFS
Katsigiannis *et al*.^21^	2019	151	28–33 days	+	15 *vs*. 17.4 *vs*. 18.2 mos	No difference in OS and PFS among 3 group, but worse OS with > 48 days
Buszek *et al*.^4^	2020	45,942	4–8 wks	67%	13.9 *vs*. 15.2 *vs*. 14.6 mos	4–8 wks has better OS, In GTR, > 8 wks has worse OS, In STR < 4 wks has worse OS
Ahn *et al*.^22^	2020	138	4 wks	+	15.5 *vs*. 14.5 mos	No differences in OS and PFS, In STR > 4 week has worse OS
Press *et al*.^17^	2020	30,414	0-8 wks	N.A.	12.8 to 16.2 mos	Worse OS in < 3 weeks No difference beyond 5 wks
Zhang *et al*.^15^	2020	66	6 wks	+	26.6 *vs*. 15.7 mos	Worse OS and PFS > 6 wks
Current study	2025	91	6 wks	+	18 *vs*. 19 mos	No differences in OS and PFS

1 GB = glioblastoma; GTR = gross-total resection; mos = months; N.A: = not available; OS = overall survival; PFS = progression-free survival; STR = subtotal resection; TMZ = temozolomide; wks = weeks

In conclusion, our study found that delays in adjuvant CRT did not affect either OS or PFS. Performance score and the type of surgical resection were identified as the most critical prognostic factors for survival. Despite being a highly aggressive tumor, the interval between surgery to CRT in GB patients with good performance status may be negligible. However, further studies are needed to evaluate the effects of delayed adjuvant therapy in patients with subtotal resections.
